# Isometric versus isotonic exercise for greater trochanteric pain syndrome: a randomised controlled pilot study

**DOI:** 10.1136/bmjsem-2019-000558

**Published:** 2019-09-21

**Authors:** Christopher Clifford, Lorna Paul, Grant Syme, Neal L Millar

**Affiliations:** 1 Department of Physiotherapy, NHS Greater Glasgow and Clyde, Glasgow, UK; 2 Institute of Infection, Immunity and Inflammation, College of Medical Veterinary and Life Sciences, University of Glasgow, Glasgow, UK; 3 School of Health and Life Sciences, Glasgow Caledonian University, Glasgow, UK; 4 Department of Physiotherapy, NHS Fife, Kirkcaldy, UK

**Keywords:** Tendinopathy, Physiotherapy, Tendinosis, Hip

## Abstract

**Objectives:**

Greater trochanteric pain syndrome (GTPS) is a common cause of lateral hip pain. Limited evidence exists for the effectiveness of exercise for GTPS. This study aimed to compare the effectiveness of isometric and isotonic exercise for individuals with GTPS.

**Methods:**

This randomised controlled pilot trial recruited 30 participants with GTPS. Both programmes consisted of daily, progressive home exercise for 12 weeks with 8 individual physiotherapy sessions over the trial period. The primary outcome measure was the Victorian Institute of Sport Assessment-Gluteal (VISA-G) and secondary outcome measures included the Numeric Pain Rating Scale (0–10) and an 11-point Global Rating of Change Scale. Outcome measures were assessed at baseline, 4 and 12 weeks.

**Results:**

Twenty-three participants completed the trial. After 12 weeks, mean VISA-G scores improved in both groups; 55–65 in the isometric group and 62–72 in the isotonic group. 55% of the isometric group and 58% of the isotonic group achieved a reduction in pain of at least 2 points (minimally clinically important difference (MCID)) on the Numeric Pain Rating Scale. 64% of the isometric group and 75% of the isotonic group had improved by at least 2 points (MCID) on the Global Rating of Change Scale.

**Conclusion:**

Isometric and isotonic exercise programmes appear to be effective for individuals with GTPS and should be considered in the loading management of patients with this condition.

**What are the new findings?**

- This is first study to compare isometric and isotonic exercise over a period of 12 weeks for greater trochanteric pain syndrome (GTPS).
- No difference was found between groups after 12 weeks over various outcomes.
- Isometric and isotonic exercise programmes both appear to be effective in reducing pain and improving function for GTPS.
- After 12 weeks over 35% of patients in both groups did not improve.

**How might it impact on clinical practice in the future?**

- Although larger trials are required, both isometric and isotonic exercise programmes can benefit patients with GTPS.
- Isometric exercise programmes of >4 weeks’ duration should be investigated for other tendinopathies.
- Future research should explore why some patients with GTPS do not appear to respond to loading programmes.

## Introduction

Greater trochanteric pain syndrome (GTPS) encompasses a number of conditions which can cause pain around the greater trochanter of the femur.^1^ The pathology primarily involves the gluteus medius and minimus tendons and less frequently the trochanteric bursa.^2 3^ Gluteal tendinopathy is the most common tendon pathology affecting the lower limb in a primary care population with incidence being as high as 24% in females and 9% in males aged 50–79 years.^4 5^ Sleep, sick leave and participation in sport are impacted with quality of life scores similar to those with severe hip osteoarthritis.^6 7^

Despite its prevalence and influence on quality of life, only three studies have investigated the effectiveness of an exercise programme for the management of GTPS. In one study, a 12-week programme of hip stretching and strengthening exercises was compared with shock wave therapy and a corticosteroid injection.^8^ Exercise was shown to be less effective after 1 month, but more beneficial at 15 months. Recently, a progressive lower limb exercise programme for the gluteal, quadriceps and calf muscles was compared with sham exercise in postmenopausal females with GTPS. Both groups also received education and similar improvements were observed at 52 weeks.^9^ A randomised controlled trial of 204 participants with gluteal tendinopathy found that education plus a progressive exercise programme targeting the gluteal muscles was superior to both a corticosteroid injection and ‘wait and see’ approach at 8 and 52 weeks.^10^

Isometric exercises have gained popularity in recent years in the management of other lower limb tendinopathies.^11^ An isometric exercise programme was compared with an isotonic programme in Australian volleyball and basketball players with patella tendinopathy.^12^ Both programmes were equally effective after 4 weeks in reducing pain and improving function, possibly indicating that the specific muscle contraction type may be less important than the loading intensity. Furthermore, a systematic review examining tendon adaptation in response to exercise concluded that loading magnitude and muscle contraction intensity was more important than muscle contraction type.^13^

Exercise is currently the first-line treatment for tendinopathy and at least 12 weeks of progressive loading is recommended.^14^ However, a recent review noted the lack of consensus around exercise and rehabilitation protocols for GTPS.^15^ Isometric and isotonic exercise programmes have not been directly compared for GTPS and it is unclear whether the improvements observed in other lower limb tendinopathies could be replicated in GTPS.

This is the first trial to compare isometric exercise with isotonic exercise, with similar magnitude of load for individuals with GTPS. Our primary aim was to evaluate and compare the outcomes for individuals with GTPS who complete a 12-week exercise programme of either progressive isometric or progressive isotonic exercises. Thus, the null hypothesis was that there would be no difference in our primary end point (Victorian Institute of Sport Assessment-Gluteal (VISA-G)) between the groups performing isometric and isotonic exercise at 12 weeks.

## Method

### Study design

This pilot randomised controlled trial compared 12 weeks of daily, progressive home-based isometric exercise with 12 weeks of daily, progressive home-based isotonic exercise for patients with greater trochanteric pain syndrome. The Consolidated Standards of Reporting Trials 2010 checklist was used to report the study.^16^ The trial was prospectively registered at www.clinicaltrials.gov NCT03145233. Patients were not directly involved in the design of this pilot study. The results were disseminated to all participants following the completion of the manuscript.

### Participants

**Figure 1:**
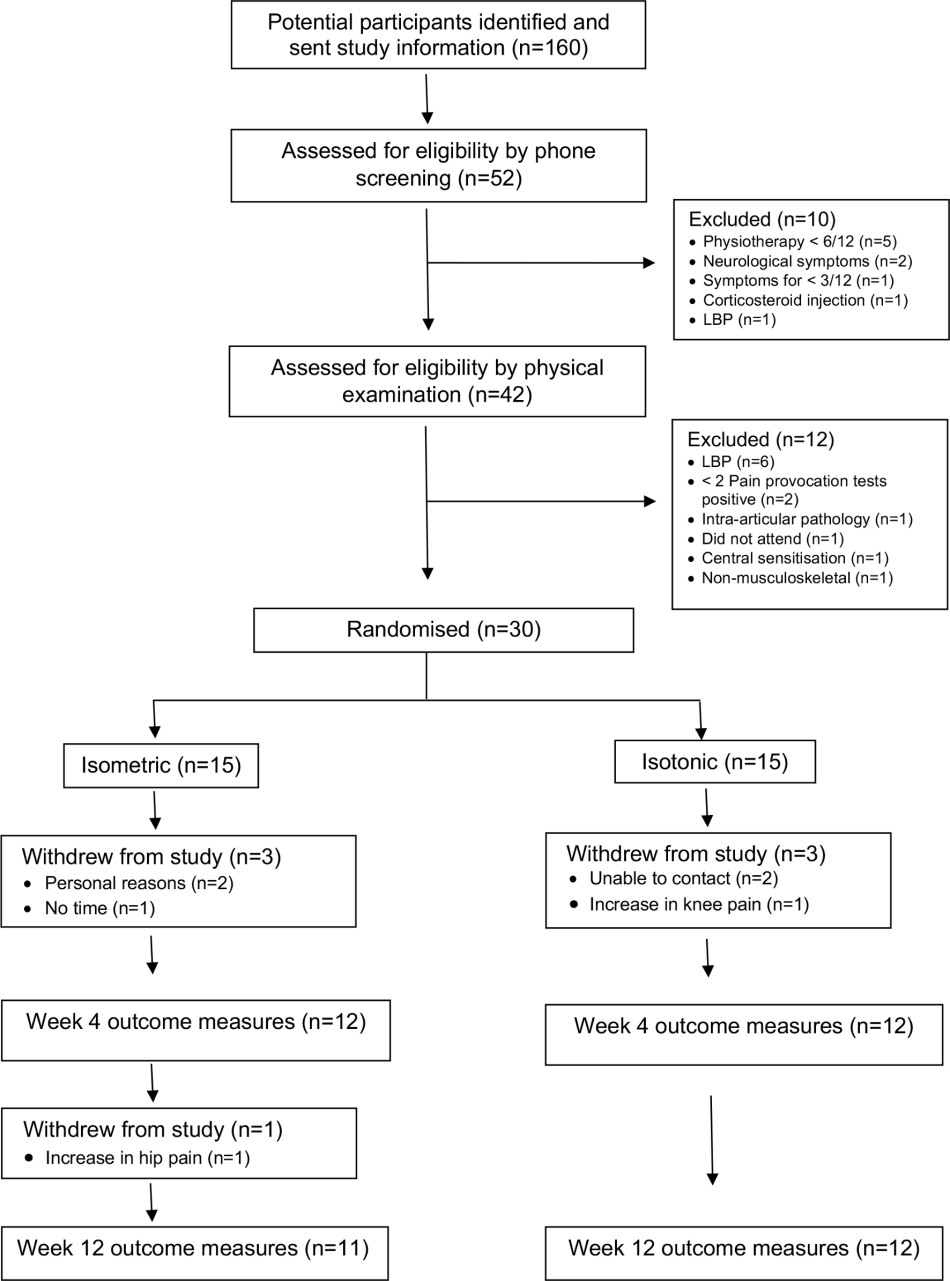
Flow diagram of participants through study. LBP, lower back pain.

Thirty participants were recruited from physiotherapy waiting lists in NHS Greater Glasgow and Clyde between August 2017 and March 2018 (figure 1). As this is a pilot study, the sample size was decided pragmatically based on the number of patients referred to physiotherapy each month with GTPS.

Individuals with lateral hip pain or a provisional diagnosis of gluteal tendinopathy and/or trochanteric bursitis were sent study information. Telephone screening and a physical examination were used to confirm a clinical diagnosis of GTPS. If hip joint pathology was suspected, a pelvis X-ray was requested. Appointments took place within the physiotherapy department at West Glasgow Ambulatory Care Hospital.

To be eligible for inclusion participants had to be aged >18 years, have lateral hip pain for >3 months, have lateral hip pain on direct palpation around the greater trochanter with pain also reproduced in at least one other of five pain provocation tests described previously by Grimaldi *et al.*^17^

Participants were excluded if they had physiotherapy for lateral hip pain in the previous 6 months, had received a corticosteroid injection for lateral hip pain in the previous 3 months, were unable to actively abduct the affected hip in side-lying, had pain reproduced with flexion, adduction, internal rotation of the hip with concurrent hip osteoarthritis on anterior posterior pelvis radiographs defined as Kellgren-Lawrence >grade 2 (mild), had previous hip or lumbar spine surgery in the previous 12 months or other medical conditions which could affect their ability to participate in the study.

### Randomisation and blinding

After giving written informed consent participants were randomly assigned into either the isometric or the isotonic exercise group. Sealed opaque envelopes were used; 15 envelopes contained labels inside with the word ‘isometric’ and 15 ‘isotonic’. Each consecutive participant selected an envelope from a box which contained all of the envelopes. Participant screening, enrolment, examination and outcome measure assessments were completed by the chief investigator (CC).

### Interventions

The isometric and isotonic exercise programmes consisted of daily exercise for 12 weeks. Daily loading for 12 weeks has been used previously for other lower limb tendinopathies with positive outcomes.^18 19^ Both programmes were designed to target the gluteus medius and minimus muscles. The exercises chosen have previously been shown to exhibit high levels of electrical muscle activity as measured by electromyography making them appropriate for muscle strengthening and tendon loading.^20–22^ A maximum of 5/10 on the Numeric Pain Rating Scale (NPRS) was allowed during exercise as long as this eased afterwards and did not increase during the night or the following day. No external resistance was used initially, but progressive muscle and tendon loading was achieved through the introduction of progressive therapeutic elastic bands ranging from low to high resistance, which induce higher levels of muscle activity and tendon loading. Exercise progression with the resistance bands was individualised and based on each participant’s ability to complete the exercises without increasing their pain beyond 5/10. All bands were 100 cm in length and were attached around both ankles.

### Isometric exercise programme

**Figure 2:**
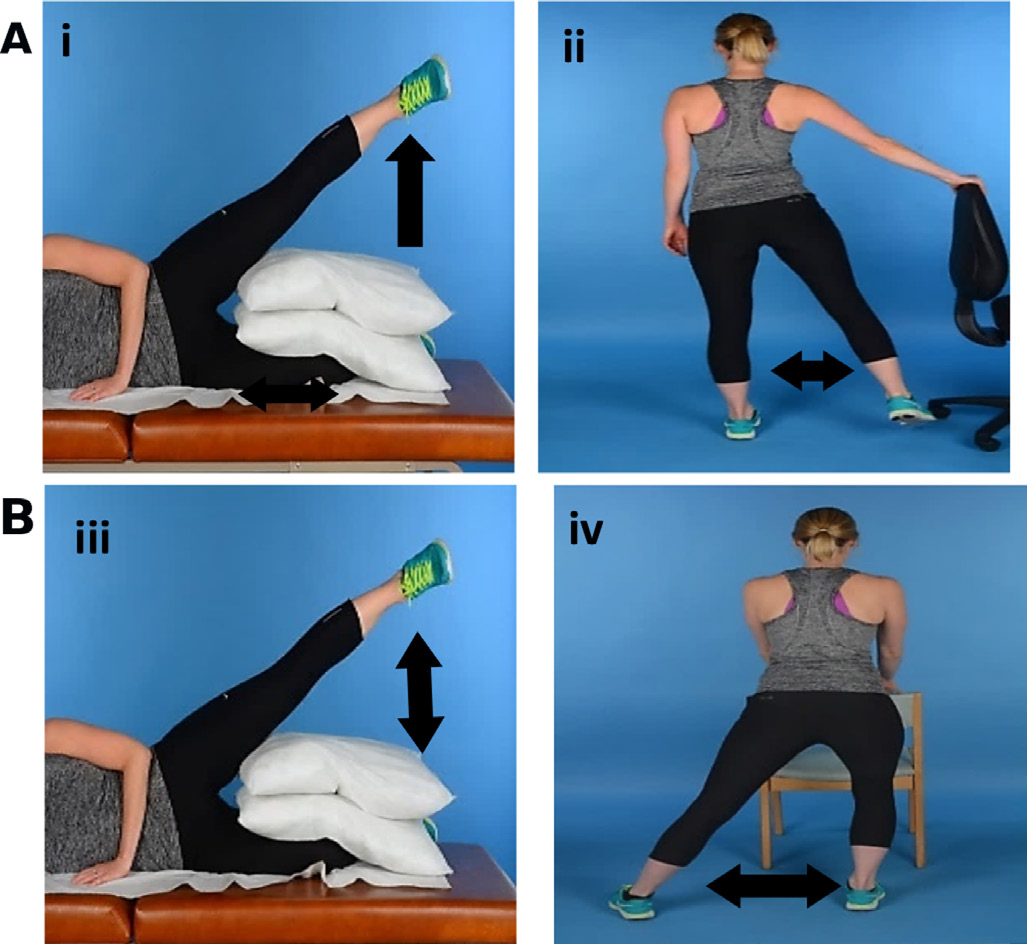
Loading programmes. (A) Isometric exercise programme: (i) hip abduction hold; (ii) weight-bearing gluteal contraction. The left leg is the affected side. (B) Isotonic exercise programme: (iii) side-lying hip abduction; (iv) hip abduction slide.

The isometric exercise programme consisted of two exercises (figure 2A and Supplementary Figure 1). The hip abduction hold (i) was completed while lying on the non-affected side with pillows between both knees. The affected hip was abducted to approximately 30 degrees in mid-line abduction and held for 30 s while maintaining knee extension. This was completed six times with 60 s rest between each repetition. During the weight-bearing gluteal contraction exercise (ii), the participant while holding onto a wall or chair for support, moved the unaffected hip through abduction/adduction to the count of 6 s, achieving an isometric gluteal contraction of the weight-bearing leg. Three sets of 10 repetitions were completed with 60 s rest between each set. Time under tension (TUT), the total time in which the muscle/tendon unit is under load during exercise was 6 min daily.

### Isotonic exercise programme

The isotonic exercise programme also consisted of two exercises (figure 2B and Supplementary Figure 2). Side-lying hip abduction (iii) was completed while lying on the non-affected side with pillows between both knees. The affected hip was abducted to approximately 30 degrees in mid-line abduction and then lowered while maintaining knee extension. The hip abduction slide (iv) is similar to an exercise used in a recent study.^10^ It was completed in upright standing with both hands supported on a chair or table. The affected leg moved into hip abduction while keeping the foot in contact with the floor and maintaining knee extension. The non-affected hip and knee could bend to around 45 degrees. The foot then slides back into the starting position. Both exercises were completed for 3 sets of 10 repetitions with 60 s rest between each set. Each repetition was 6 s duration (3 s concentric, 3 s eccentric). TUT was 6 min daily.

All participants attended eight individual physiotherapy appointments during the 12-week programme. All sessions were with the chief investigator, a physiotherapist with 11 years of clinical experience with musculoskeletal disorders and a postgraduate master degree-level qualification. During the first appointment, participants were given the opportunity to practice their exercise programme under supervision of the chief investigator to ensure correct technique. Following this, participants attended weekly for the next 2 weeks and thereafter for a further five sessions over the next 10 weeks to ensure correct exercise technique and for exercise progression. Postural education and advice on positions that could be used to reduce pain during daily activities were also given. An illustrated instructional exercise booklet was provided. A daily exercise diary was used to monitor exercise adherence and to record the number of repetitions completed and the maximum pain score elicited during the exercise protocols. Simple analgesia was permitted, but participants were asked to refrain from seeking other forms of treatment during the study. Participants were also encouraged to remain physically active within their limits of pain.

### Outcomes

Outcome measures were assessed at baseline, 4 and 12 weeks. The primary outcome measure was the VISA-G Questionnaire, which has been validated for use in patients with GTPS.^23^ It consists of eight questions assessing current symptoms with total scores ranging from 0 to 100. Higher scores indicate less pain and better function.

The secondary outcome measures were the following:

- The NPRS is a unidimensional measure of the average pain intensity the previous week.^24^ It is measured on an 11-point scale between 0 (no pain at all) and 10 (worst pain imaginable).
- The Global Rating of Change (GROC) Scale was used to assess perceived overall change in lateral hip pain. An 11-point Likert scale ranging from ‘very much worse’ to ‘completely recovered’ was used.^25^
- The Pain Catastrophising Scale (PCS) consists of 13 statements.^26^ Participants indicated on a 5-point scale the degree to which they had certain thoughts and feelings when they were experiencing pain. A rating of 0 (not at all) to 4 (all the time) can be given. The total scores range from 0 to 52 with higher scores indicating higher levels of pain catastrophisation.
- The Hip Disability and Osteoarthritis Outcome Score (HOOS) consists of five subscales: symptoms/stiffness, pain, function in activities of daily living, function in sport and recreation and quality of life.^27^ Each questions has five possible answers, scored from 0 to 4. Total scores of 0 indicates a severe problem and 100 which would indicate no problem.
- The Euro Qol (EQ-5D-5L) is a five-dimension questionnaire and a standardised instrument for measuring generic health status.^28^ Health status is measured in terms of five dimensions (5D): mobility, self-care, usual activities, pain/discomfort and anxiety/depression. Each of these five dimensions has five statements and each participant was asked to tick one of these five boxes for each dimension. The participant also evaluated their own current overall health status using the Visual Analogue Scale with a score of 0 indicating the worst health they can imagine and a score of 100 the best health they can imagine.
- The International Physical Activity Questionnaire Short Form (IPAQ-SF) measures physical activity.^29^ The seven questions relate to the amount of time the participant has spent being physically active in the previous 7 days. Results can be reported as either low, moderate or high activity levels or separately as a single numerical value based on the amount of energy expended during physical activity.

### Statistical analysis

Statistical analysis was performed using Minitab (V.18). Data were found to be normally distributed. Descriptive statistics were used to describe the sample and the trends in the data over time for both groups. The groups were compared at 4 and 12 weeks using means and 95% CIs. Cohen’s d effect sizes were calculated for the VISA-G using a threshold of 0.2 (small), 0.5 (medium) and 0.8 (large).^30^ Per-protocol analysis was undertaken, and statistical significance taken as p<0.05.

## Results

### Participants

Thirty participants with GTPS were randomised into isometric and isotonic groups. Group characteristics were found to be comparable at baseline (table 1). Twenty-three participants were included in the final analysis. A total of seven participants did not complete the study (figure 1). One participant in the isometric group withdrew due to an increase in hip pain and in the isotonic group one participant withdrew due to an increase in knee pain. The other five withdrawals were due to reasons unrelated to the study. One participant in the isotonic group sustained an injury not related to the study and reported an increase in symptoms.

**Table 1:** Participant characteristics (mean (SD) unless otherwise stated)

	Isometric (n=15)	Isotonic (n=15)
Age (years)	57.5 (16.8)	61.1 (15.2)
Female	13	14
Height (cm)	164.4 (7.0)	159.1 (8.9)
Weight (kg)	74.1 (11.7)	75.4 (17.6)
Body mass index (kg/m^2^)	27.7 (4.1)	29.6 (4.8)
Duration of symptoms (months)	23 (21.4)	22.9 (28.3)
Unilateral symptoms	13	13
Previous steroid injection	7	2
Low back pain	8	10
Groin pain	3	4
Diabetes	2	3

### Primary outcome

#### Victorian Institute of Sport Assessment-Gluteal

**Figure 3:**
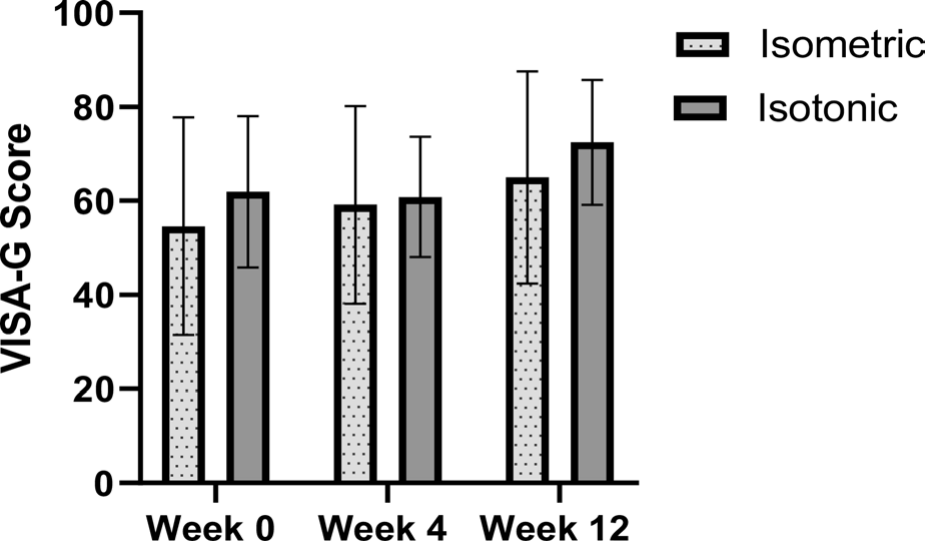
Victorian Institute of Sport Assessment-Gluteal (VISA-G) scores at 0, 4 and 12 weeks following treatment.

Both groups had similar improvements in VISA-G scores over the course of the intervention period (figure 3). The isometric group increased from a mean of 54.6±23.1 points at baseline to 59.2±21.0 (week 4) to 65.0±22.6 (week 12). The isotonic group scored a mean of 61.9±16.1 at baseline, 60.8±12.8 (week 4) and 72.4±13.3 (week 12). At week 4 between group differences were 5.5 points (95% CI −3.5 to 14.4) and −0.1 points (95% CI −13.8. to 13.5) at week 12. Effect sizes at week 12 were d=0.45 (isometric) and d=0.71 (isotonic).

### Secondary outcomes

#### Numeric Pain Rating Scale

**Figure 4:**
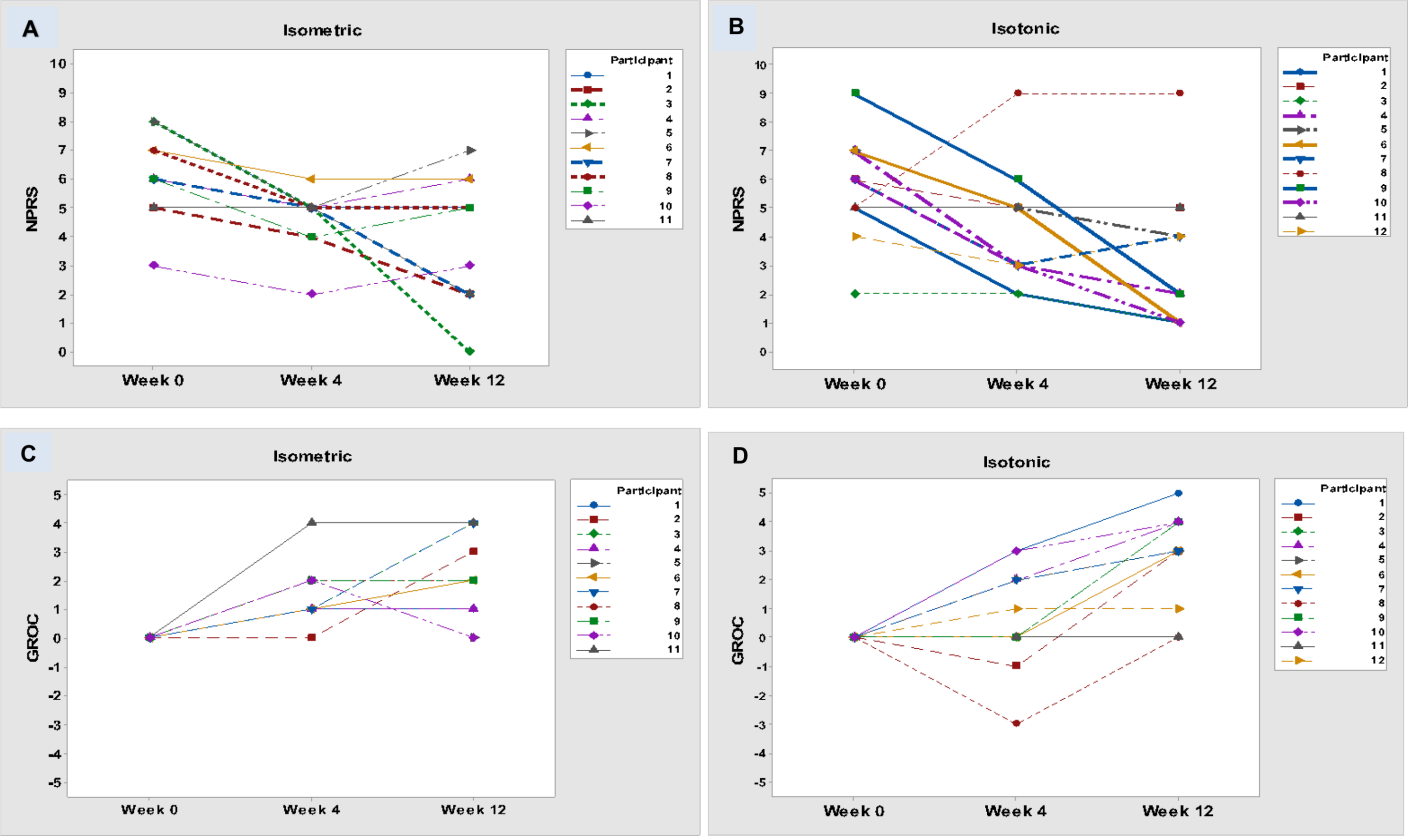
Numeric Pain Rating Scale (NPRS): (A) isometric group, (B) isotonic group. Global Rating of Change (GROC) Scale: (C) isometric group, (D) isotonic group. *Note*: due to the same scores reported for different participants, some lines overlap.

In the isometric group, 5/11 (45%) achieved a pain reduction by the minimally clinically important difference (MCID) of at least 2 points by week 4, compared with 7/12 (58%) of the isotonic group (figure 4). By week 12, 6/11 (55%) of the isometric group and 7/12 (58%) of the isotonic group had achieved the MCID.

#### Global Rating of Change Scale

At week 4, 5/11 (45%) of participants in both groups had improved by the MCID of 2 points (figure 4); 7/11 (64%) of the isometric group and 9/12 (75%) of the isotonic group reported a meaningful change by week 12.

For the remainder of the secondary outcome measures there was no significant difference between groups at both 4 and 12 weeks (table 2). For the PCS, mean scores reduced by 2.9 points in the isometric group by week 4 and 3 points by week 12. Scores in the isotonic group improved by 1.8 points by week 4 and 6.3 points by week 12. All domains improved in both groups for the HOOS with trends towards statistically significant findings in the isotonic group for both pain and quality of life. For the EQ-5D-L, there were minimal changes in both index and health scores. For the IPAQ-SF, there was minimal change in levels of physical activity over the course of the 12 weeks.

**Table 2:** Secondary outcome measures

Outcome	Group mean scores and (SD)						Difference within groups (95% CI)				Difference between groups (95% CI)	
	0 weeks		4 weeks		12 weeks		0–4 weeks		0–12 weeks		4 weeks	12 weeks
	Isometric	Isotonic	Isometric	Isotonic	Isometric	Isotonic	Isometric	Isotonic	Isometric	Isotonic		
	(n=11)	(n=12)	(n=11)	(n=12)	(n=11)	(n=12)	(n=11)	(n=12)	(n=11)	(n=12)		
PCS (0–52)	10.3 (10.7)	13.0 (13.6)	7.4 (7.2)	11.2 (10.4)	7.3 (9.8)	6.8 (8.6)	2.9 (5.2 to 11)	1.8 (8.4 to 12.1)	3.0 (6.1 to 12.1)	6.3 (3.4 to 15.9)	0.7 (−4.4 to 5.8)	−3.3 (−12.8 to 6.3)
							p=0.462	p=0.714	p=0.500	p=0.193	p=0.777	p=0.487
HOOS (0–100)												
Symptoms	60.9 (15.6)	65.0 (18.7)	67.3 (14.2)	64.2 (17.8)	72.7 (19.8)	76.7 (12.5)	6.4 (−6.9 to 19.6)	−0.8 (−16.3 to 14.6)	11.8 (−4.0 to 27.7)	11.7 (−1.8 to 25.3)	8.7 (−11.4 to 28.7)	0.2 (−19.3 to 19.6)
							P=0.329	P=0.912	P=0.136	P=0.086	P=0.378	P=0.987
Pain	53.6 (15.8)	53.8 (16.8)	61.1 (15.5)	59.8 (15.9)	65.7 (20.2)	70.7 (24.3)	7.5 (−6.4 to 21.4)	6.0 (−7.0 to 19.1)	12.0 (−3.6 to 27.6)	17.0 (0.7 to 33.3)	−0.3 (−17.1 to 16.4)	−3.4 (−27.0 to 20.2)
							P=0.274	P=0.351	P=0.125	**P=0.042**	P=0.966	P=0.768
Function/daily living	59.4 (18.4)	64.0 (17.4)	68.5 (18.3)	68.9 (17.8)	72.56 (19.7)	73.0 (26.8)	9.1 (−7.7 to 25.8)	4.9 (−9.1 to 18.9)	13.2 (−3.7 to 30.1)	9.0 (−8.6 to 26.5)	0.7 (−18.9 to 20.3)	1.6 (−23.1 to 26.4)
							P=0.272	P=0.480	P=0.144	P=0.303	P=0.937	P=0.890
Sports	51.1 (26.4)	60.0 (20.8)	60.4 (23.6)	61.0 (17.7)	63.8 (23.2)	68.8 (28.9)	9.3 (−13.5 to 32.2)	1.0 (−15.5 to 17.5)	12.7 (−10.2 to 35.6)	8.7 (−12.3 to 29.8)	10.2 (−9.0 to 29.4)	7.2 (−14.8 to 29.1)
							P=0.402	P=0.899	P=0.260	P=0.398	P=0.280	P=0.502
QoL	44.3 (22.6)	50.0 (16.0)	51.7 (12.5)	50.0 (20.0)	56.8 (20.4)	67.2 (19.2)	7.6 (−7.6 to 22.8)	0.0 (−14.6 to 14.6)	12.5 (−6.3 to 31.3)	17.2 (3.2 to 31.2)	5.6 (−11.6 to 22.7)	−10.7 (−28.9 to 7.4)
							P=0.313	P=1.000	P=0.180	**P=0.018**	P=0.503	P=0.231
EQ-5D-5L												
Index (0–1)	0.64 (0.19)	0.63 (0.15)	0.69 (0.14)	0.62 (0.17)	0.70 (0.15)	0.69 (0.18)	0.05 (−0.104 to 0.194)	−0.01 (−0.147 to 0.128)	0.06 (−0.089 to 0.217)	0.07 (−0.076 to 0.201)	0.040 (−0.063 to 0.142)	−0.03 (−0.132 to 0.125)
							P=0.535	P=0.887	P=0.397	P=0.343	P=0.435	P=0.960
Health score (0–100)	66.8 (13.8)	70 (19.3)	66.8 (15.4)	69.2 (16.5)	70.5 (15.7)	72.1 (20.5)	0.0 (−13.0 to 13.0)	−0.8 (−16.0 to 14.34)	3.6 (−9.5 to 16.8)	2.1 (−14.8 to 18.9)	0.8 (−13.7 to 15.4)	1.6 (−18.6 to 21.7)
							P=1.000	P=0.911	P=0.571	P=0.800	P=0.906	P=0.871
IPAQ-SF												
Low	4	3	3	3	4	4						
Moderate	4	5	7	4	3	3						
High	3	4	1	4	3	4						
				(one missing)	(one missing)	(one missing)						

EQ-5D-5L, 5-level Euro Qol; HOOS, Hip Disability and Osteoarthritis Outcome Score; IPAQ-SF, International Physical Activity Questionnaire Short Form; PCS, Pain Catastrophising Scale; QoL, quality of life.

#### Adherence

All participants, except one in the isometric group completed exercise diaries. Hundred per cent of participants who finished the trial completed at least 50% of the daily exercise sessions. Seven of the 10 (70%) of the isometric group completed at least 80% of the sessions compared with 7/12 (58%) of the isotonic group. Eighty per cent adherence has recently been suggested to be a reasonable threshold in exercise intervention studies.^31^ All participants, except one in the isometric group, were able to progress the loading intensity of the exercises and used resistance bands.

## Discussion

This is the first study to compare isometric and isotonic exercise for greater trochanteric pain syndrome. Both groups showed similar improvements over a number of outcomes when compared at both 4 and 12 weeks. We found that mean VISA-G scores improved by just over 10 points in both groups by the end of the study. The MCID for the VISA-G has yet to be determined so it is unclear if these improvements are clinically significant. The percentage of participants who reported a reduction in pain of at least 2 points (MCID) on the NPRS was similar between groups at 12 weeks. For GROC, there were equal improvements in both groups at 4 weeks, with both groups demonstrating further increases in the number of participants reporting an MCID of 2 points at 12 weeks. This was more apparent in the isotonic group with a higher percentage reporting a clinically important change. Small improvements were seen in both groups for pain catastrophising, but only one participant in each group had a score of >30 at baseline suggesting that catastrophising is uncommon in this patient population. Improvements for both groups were found across all domains of the HOOS. No meaningful differences were detected for physical activity and health status when measured by the EQ-5D-5L and IPAQ-SF, respectively, which is unsurprising given the relatively short intervention period and follow-up. This may be due to the study not been sufficiently powered to detect changes in these measures.

Only a small number of studies have investigated isometric exercise in lower limb tendinopathy with mixed reported benefits on pain outcomes.^32–36^ The current findings support previous results in athletes with patella tendinopathy,^12^ where no differences in pain or VISA-G scores were found after a 4-week exercise programme of isometric or isotonic exercise. In the present study, both groups were similar when compared at 12 weeks. In both studies, isometric and isotonic groups were matched for TUT and muscle contraction type, although our study used 30 s contractions compared with 45 s. Hip abductor weakness is known to be present in patients with GTPS,^32^ and in clinical practice patients are often unable to maintain an isometric hip abduction contraction for 45 s in side-lying due to pain or strength deficits. However, isometric contractions sustained for 30 s were well tolerated by participants during our study. Regardless, the duration of the isometric contraction does not appear to have a significant effect on the outcome with long and short holds being equally effective.^34^

Three previous studies have investigated exercise for GTPS. The ‘LEAP’ trial reported an increase of around 19 points for the VISA-G in the education and exercise group after 12 weeks.^10^ Similar to our study, participants received advice and education on tendon care, both exercise programmes were progressive and pain was permitted during exercise. However, our trial had two exercises per group and the LEAP study had four to six exercises throughout the duration of the 8-week programme so weekly TUT is likely to have been of longer duration. The LEAP study also excluded participants who reported either low back pain or groin pain of >2/10 on the NPRS. We decided not to exclude these participants as concurrent low back and/or groin pain is common in GTPS, with low back pain prevalence being as high as 35%.^37^ At the beginning of our study 10 participants (33%) reported low back pain >2/10 (NPRS) and 7 participants (23%) experienced groin pain >2/10. It is possible that patients with concurrent low back pain and/or groin pain do not respond as favourably to a targeted gluteal strengthening programme and may require additional treatment which specifically addresses these areas of pain. Further research is required to investigate whether a combined management programme focusing on both GTPS and low back and/or groin pain would lead to better outcomes.

The ‘GLoBE’ trial compared gluteal loading with sham exercises in postmenopausal women with GTPS.^9^ Both groups received education on activity modification and avoiding tendon compression. After 12 weeks, VISA-G scores of both groups improved by around 11 points when analysed by intention-to-treat, which is similar to our study, although we analysed by per-protocol. We used a similar education programme and it is possible that the improvements observed in both studies could be attributed to the education component of the management programme.

Rompe *et al*^8^ used a 12-week exercise programme, which included daily strengthening and stretching exercises. Results highlighted that only 7% of participants improved after 4 weeks, which increased to 41% after 4 months on a GROC Scale. More recent evidence has highlighted the importance of minimising compression in tendinopathy management.^38^ It is possible therefore that previous stretching programmes may have actually increased pain through compression of the gluteal tendons and bursa, which would explain the lack of initial improvement. We found that 45% of the participants reported a meaningful change after 4 weeks on the GROC, which increased to 64% in the isometric group and 75% in the isotonic group after 12 weeks. Both the LEAP and GLoBE trials highlighted the importance of reducing compressive loading and this appears to be supported by the results of the present study.

Isometric and isotonic exercise programmes appear to have similar effects on tendinopathy regardless of muscle contraction type. A systematic review concluded that muscle contraction intensity and not contraction type was more important in tendinopathy loading programmes.^13^ This is supported by the results of the present study. Prescribing either isometric or isotonic exercise appears to lead to clinical improvements. Isometric exercise programmes similar to the one used in the present study may be useful in certain patient populations, for example, GTPS and concurrent moderate-to-severe hip osteoarthritis as these patients typically exhibit reduced range of motion and are unable to complete isotonic exercises that involve wide-range hip abduction movements.

Almost all participants, in both groups, who had a reduction in pain of at least two points on the NPRS at week 12 had already achieved this by week 4. It is possible that the education component of the programme was responsible for this as positive initial improvements were also reported in the LEAP trial. Although, muscle hypertrophy and tendon adaptation will not occur within this timeframe in response to resistance exercise, neuromuscular adaptation can occur quickly and may explain this early improvement in symptoms.

As exercise is the first-line treatment for tendinopathy, it is important to explore and improve the delivery of different types of exercise programmes. Future research could compare a combined isometric/isotonic programme with an isolated isotonic or isometric programme, or an education-only intervention to determine the optimal management approach.

Despite the benefits of both isometric and isotonic exercise, several participants in both groups did not experience improvement in symptoms by the end of the study. VISA-G scores were worse or unchanged in 4/11 (36%) of the isometric group and 5/12 (42%) of the isotonic group. In the GLoBE study, around 50% of participants reported either increased pain or no change in pain after 52 weeks. Further research is required to explore why a sizeable number of patients with GTPS, and other lower limb tendinopathies,^39^ do not respond to a targeted loading programme. Recent evidence highlights that some patients with GTPS and severe symptoms report high levels of psychological distress and poorer quality of life scores.^40^ These findings suggest that there may be subgroups of patients with GTPS who require different management strategies.

### Limitations

The results of this study are limited due to the small sample size and drop-out rate of at least 20% in each group. Despite this, group numbers included in the final analysis are comparable to other studies that have compared isometric and isotonic exercise programmes for tendinopathy.^12 41^ In the absence of a no treatment ‘control’ group, we are unable to determine if some participants improved due to the natural history of the condition. Due to available resources, we were not able to confirm the presence of gluteal tendinopathy with MRI or diagnostic ultrasound, so it is possible that participants with other pathology, for example, with partial gluteal tendon tears were included. However, this is reflective of current National Health Service clinical practice and the pain provocation tests used for inclusion have shown clinical utility and are comparable to MRI.^17^ The outcome assessor was not blinded to group allocation which introduces the potential for bias. All exercise sessions were supervised by the chief investigator, which could again introduce potential bias.

## Conclusion

This is the first randomised controlled trial comparing isometric and isotonic exercise for GTPS. Both isometric and isotonic exercise programmes appear to reduce pain and improve function. No difference was found between groups after 12 weeks of progressive exercise. Although these results need to be confirmed in a fully powered trial, it appears that the type of loading does not affect the outcome for patients with GTPS.

10.1136/bmjsem-2019-000558.supp1 Supplementary data

10.1136/bmjsem-2019-000558.supp2 Supplementary data
