# Supplementary material for: Isometric versus isotonic exercise for greater trochanteric pain syndrome: a randomised controlled pilot study
Source: BMJ Open Sport Exerc Med. 2019 Sep 21;5(1):e000558. doi: 10.1136/bmjsem-2019-000558 (PMC6797310; doi:10.1136/bmjsem-2019-000558)
Supplement: Supplementary data [file bmjsem-2019-000558supp001.pdf]

Exercise and Advice Booklet

Isometric Exercise Programme

Clifford C, *et al. BMJ Open Sp Ex Med* 2019; 5:e000558. doi: 10.1136/bmjsem-2019-000558

This booklet contains:

- Information about common postures
- The exercises that you should do each day
- An exercise diary

Background Information

The gluteal muscles are a group of muscles located in the region of the buttocks. They attach via a tendon to the bony prominence at the side of the hip. The bony prominence is called the greater trochanter.

The gluteus medius and gluteus minimus tendons are the most commonly injured tendons at the side of the hip. Compression of the gluteal tendons against the greater trochanter can occur during movements of the hip and also during certain postures and positions. This is mainly when the leg crosses the mid-line of the body.

This booklet gives advice and information on how to reduce compression of the gluteal tendons during daily activities and common postures. This is a key component of the exercise programme.

The aims of the exercise programme are to reduce pain and progressively strengthen the gluteal muscles and tendons. Initially you may need to reduce or completely stop some of your normal sports and activities, especially if your symptoms increase significantly afterwards. You can discuss this with a member of the research team.

Clifford C, *et al. BMJ Open Sp Ex Med* 2019; 5:e000558. doi: 10.1136/bmjsem-2019-000558

Common Postures

Lying

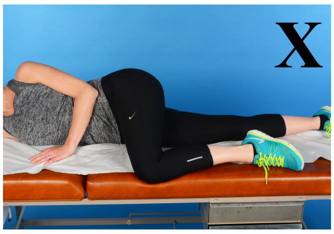
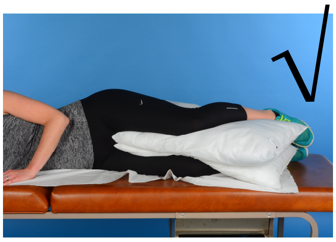
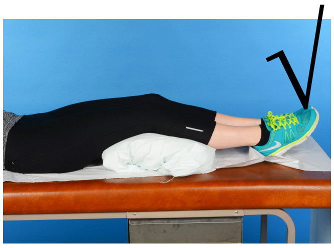

When lying down or sleeping, do not lie on the affected side. If lying on the opposite side, use pillows between your knees to keep leg in parallel position. You can also lie on your back with a pillow under your knees.

Sitting

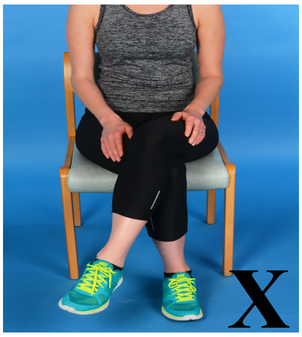
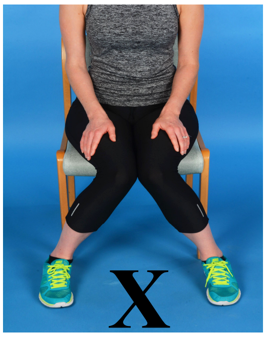
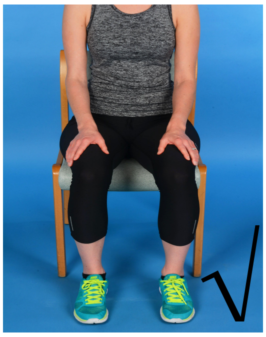

Do not cross your legs or sit with your knees together and feet apart. It may help to sit with your hips higher than your knees, you may need to use a pillow or cushion to do this or raise your chair.

Standing

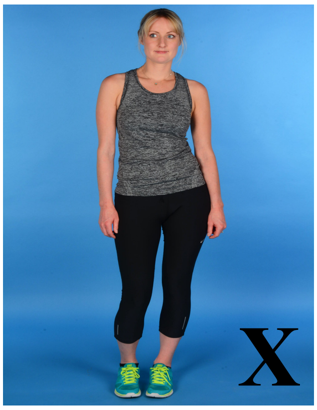
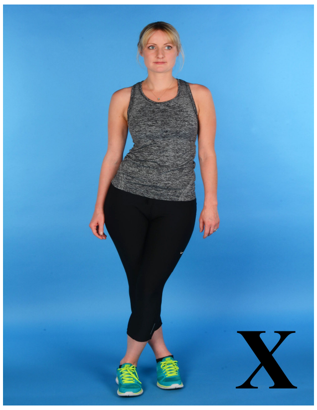
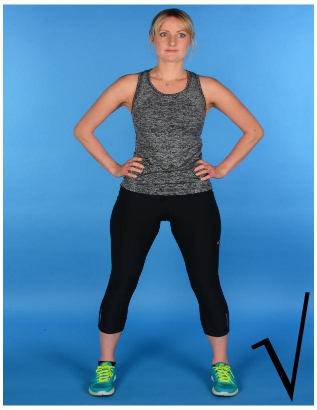

Do not stand ‘hanging on hip’ with all your weight on one leg or stand with your legs crossed. Stand upright with weight evenly shared between both legs and feet shoulder width apart.

Clifford C, *et al. BMJ Open Sp Ex Med* 2019; 5:e000558. doi: 10.1136/bmjsem-2019-000558

Rising from a chair

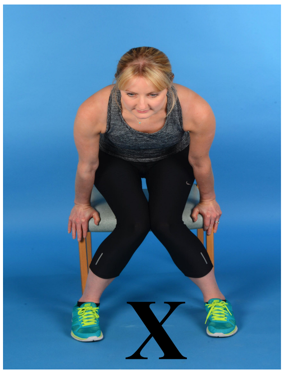

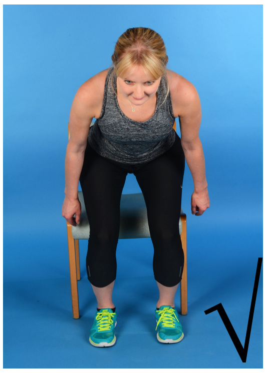

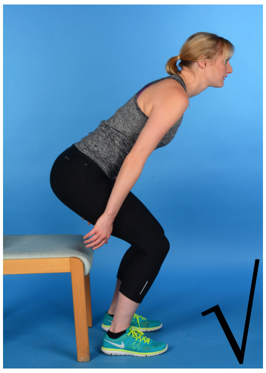

Keep knees apart. Do not allow knees to roll in across body, squeeze buttocks as you stand to help prevent this. Lean forward, bending at your hips and knees while keeping back straight.

Going up stairs

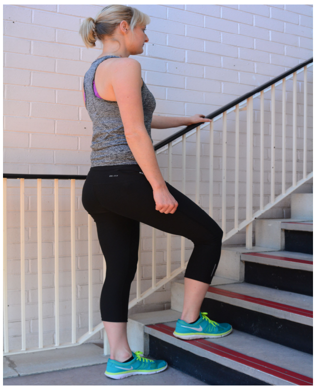

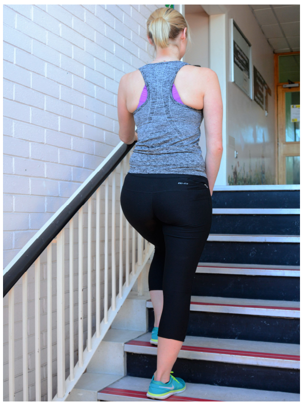

If going up stairs is painful, use a hand rail on the opposite side from your affected leg. Keep feet a little wider.

Stretching

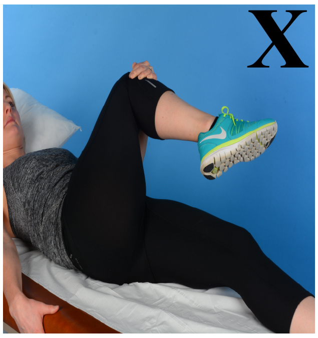

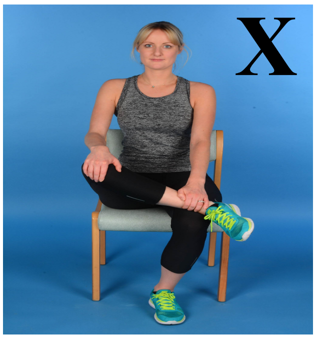

Avoid stretching your leg across your body or stretching your leg by pushing your knee down while the foot is placed on the opposite knee.

Clifford C, *et al. BMJ Open Sp Ex Med* 2019; 5:e000558. doi: 10.1136/bmjsem-2019-000558

Exercises

1. Isometric Side-Lying Hip Abduction

During isometric exercise the muscle length and position of the leg do not change. The leg is held in a static position for a set amount of time.

Starting position

Lie on your non-affected side. Bend the lower knee. Put 1 or 2 pillows between your knees so that your affected leg doesn't cross the mid-line of your body (Picture 1).

Exercise

Lift the affected leg up towards the ceiling away from the body by about 10 inches (Picture 2). This movement is called hip abduction. Do not allow this knee to bend.

Hold for 30 seconds then lower. Rest for 60 seconds between each repetition. You should feel the gluteal muscles at the side of your hip working.

How often?

Repeat 6 times.

Complete once a day

Progress to using a resistance band around both ankles when advised by your physiotherapist.

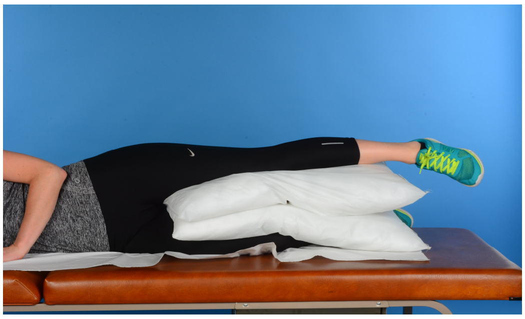

Picture 1

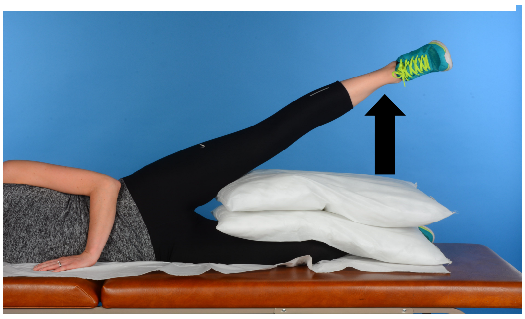

Picture 2

Clifford C, *et al. BMJ Open Sp Ex Med* 2019; 5:e000558. doi: 10.1136/bmjsem-2019-000558

2. Isometric Standing Hip Abduction

Starting position

Stand on affected leg with opposite hand supported (Picture 1).

Exercise

Take non-affected leg out to the side to the count of 3 seconds while keeping straight back (Picture 2). Make sure your pelvis doesn't drop on standing leg. Bring your leg back to starting position for the count of 3 seconds. Do not allow your foot to touch the floor.

How often?

Aim to complete 3 sets of 10 repetitions. Rest for 60 seconds between sets.

Complete once a day

Progress to using a resistance band around both ankles when advised by your physiotherapist.

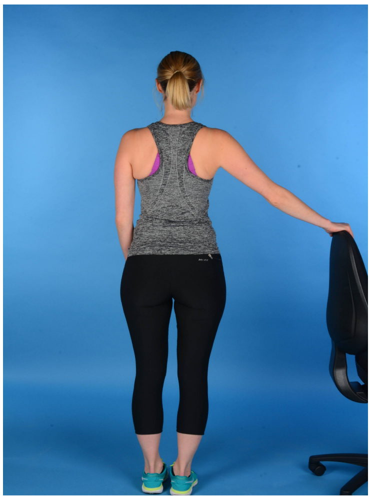

Picture 1

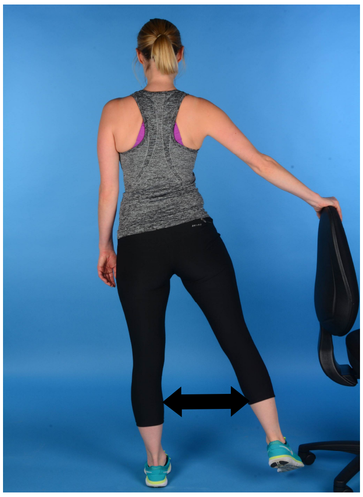

Picture 2

Clifford C, *et al. BMJ Open Sp Ex Med* 2019; 5:e000558. doi: 10.1136/bmjsem-2019-000558

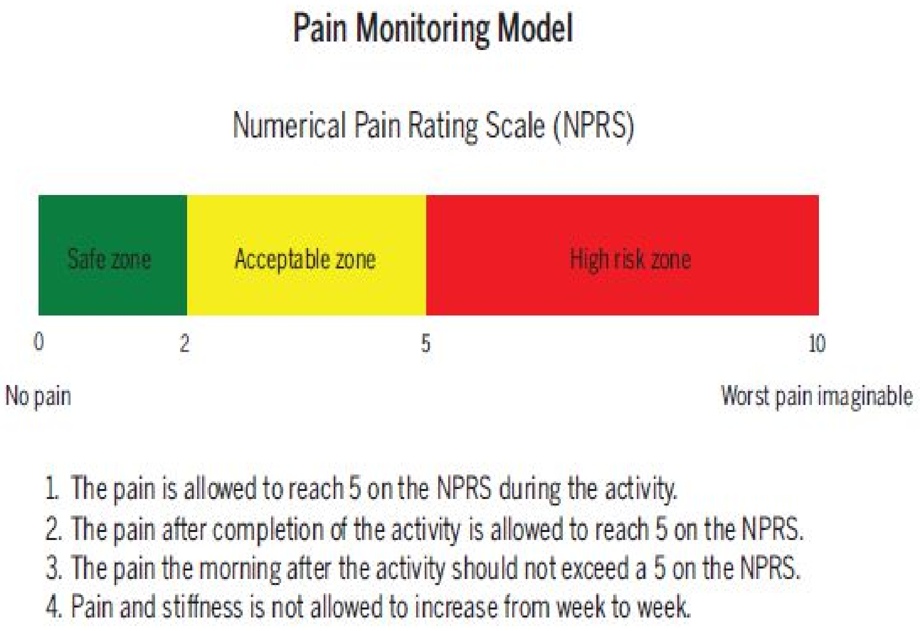

Adapted from Silbernagel 2007

You can use this pain scale can be used to monitor your pain during your exercise programme. It ranges from 0 (no pain) to 10 (worse pain imaginable). The pain is allowed to reach 5 during your exercise programme. If the pain you experience during your exercise programme is more than 5, you should reduce the number of repetitions.

University of Glasgow

NHS Greater Glasgow and Clyde

Exercise Diary

This should be completed every day for 12 weeks

Clifford C, *et al. BMJ Open Sp Ex Med* 2019; 5:e000558. doi: 10.1136/bmjsem-2019-000558

**Week 1**	Exercise 1<br>Number of repetitions completed<br>(1-6)	Exercise 2<br>Number of repetitions completed<br>(1-6)	Maximum pain score during exercise programme<br>(1-10)
Day 1			
Day 2			
Day 3			
Day 4			
Day 5			
Day 6			
Day 7			

Clifford C, *et al. BMJ Open Sp Ex Med* 2019; 5:e000558. doi: 10.1136/bmjsem-2019-000558

**Week 2**	Exercise 1<br>Number of repetitions completed<br>(1-6)	Exercise 2<br>Number of repetitions completed<br>(1-6)	Maximum pain score during exercise programme<br>(1-10)
Day 1			
Day 2			
Day 3			
Day 4			
Day 5			
Day 6			
Day 7			

**Week 3**	Exercise 1<br>Number of repetitions completed<br>(1-6)	Exercise 2<br>Number of repetitions completed<br>(1-6)	Maximum pain score during exercise programme<br>(1-10)
Day 1			
Day 2			
Day 3			
Day 4			
Day 5			
Day 6			
Day 7			

Clifford C, *et al. BMJ Open Sp Ex Med* 2019; 5:e000558. doi: 10.1136/bmjsem-2019-000558

**Week 4**	Exercise 1<br>Number of repetitions completed<br>(1-6)	Exercise 2<br>Number of repetitions completed<br>(1-6)	Maximum pain score during exercise programme<br>(1-10)
Day 1			
Day 2			
Day 3			
Day 4			
Day 5			
Day 6			
Day 7			

Clifford C, *et al. BMJ Open Sp Ex Med* 2019; 5:e000558. doi: 10.1136/bmjsem-2019-000558

**Week 5**	Exercise 1<br>Number of repetitions completed<br>(1-6)	Exercise 2<br>Number of repetitions completed<br>(1-6)	Maximum pain score during exercise programme<br>(1-10)
Day 1			
Day 2			
Day 3			
Day 4			
Day 5			
Day 6			
Day 7			

Clifford C, *et al. BMJ Open Sp Ex Med* 2019; 5:e000558. doi: 10.1136/bmjsem-2019-000558

**Week 6**	Exercise 1<br>Number of repetitions completed<br>(1-6)	Exercise 2<br>Number of repetitions completed<br>(1-6)	Maximum pain score during exercise programme<br>(1-10)
Day 1			
Day 2			
Day 3			
Day 4			
Day 5			
Day 6			
Day 7			

Clifford C, *et al. BMJ Open Sp Ex Med* 2019; 5:e000558. doi: 10.1136/bmjsem-2019-000558

**Week 7**	Exercise 1<br>Number of repetitions completed<br>(1-6)	Exercise 2<br>Number of repetitions completed<br>(1-6)	Maximum pain score during exercise programme<br>(1-10)
Day 1			
Day 2			
Day 3			
Day 4			
Day 5			
Day 6			
Day 7			

Clifford C, *et al. BMJ Open Sp Ex Med* 2019; 5:e000558. doi: 10.1136/bmjsem-2019-000558

**Week 8**	Exercise 1<br>Number of repetitions completed<br>(1-6)	Exercise 2<br>Number of repetitions completed<br>(1-6)	Maximum pain score during exercise programme<br>(1-10)
Day 1			
Day 2			
Day 3			
Day 4			
Day 5			
Day 6			
Day 7			

**Week 9**	Exercise 1<br>Number of repetitions completed<br>(1-6)	Exercise 2<br>Number of repetitions completed<br>(1-6)	Maximum pain score during exercise programme<br>(1-10)
Day 1			
Day 2			
Day 3			
Day 4			
Day 5			
Day 6			
Day 7			

Clifford C, *et al. BMJ Open Sp Ex Med* 2019; 5:e000558. doi: 10.1136/bmjsem-2019-000558

**Week 10**	Exercise 1<br>Number of repetitions completed<br>(1-6)	Exercise 2<br>Number of repetitions completed<br>(1-6)	Maximum pain score during exercise programme<br>(1-10)
Day 1			
Day 2			
Day 3			
Day 4			
Day 5			
Day 6			
Day 7			

Clifford C, *et al. BMJ Open Sp Ex Med* 2019; 5:e000558. doi: 10.1136/bmjsem-2019-000558

**Week 11**	Exercise 1<br>Number of repetitions completed<br>(1-6)	Exercise 2<br>Number of repetitions completed<br>(1-6)	Maximum pain score during exercise programme<br>(1-10)
Day 1			
Day 2			
Day 3			
Day 4			
Day 5			
Day 6			
Day 7			

Clifford C, *et al. BMJ Open Sp Ex Med* 2019; 5:e000558. doi: 10.1136/bmjsem-2019-000558

**Week 12**	Exercise 1<br>Number of repetitions completed<br>(1-6)	Exercise 2<br>Number of repetitions completed<br>(1-6)	Maximum pain score during exercise programme<br>(1-10)
Day 1			
Day 2			
Day 3			
Day 4			
Day 5			
Day 6			
Day 7			

Clifford C, *et al. BMJ Open Sp Ex Med* 2019; 5:e000558. doi: 10.1136/bmjsem-2019-000558
