# Supplementary material for: Isometric versus isotonic exercise for greater trochanteric pain syndrome: a randomised controlled pilot study
Source: BMJ Open Sport Exerc Med. 2019 Sep 21;5(1):e000558. doi: 10.1136/bmjsem-2019-000558 (PMC6797310; doi:10.1136/bmjsem-2019-000558)
Supplement: Supplementary data [file bmjsem-2019-000558supp002.pdf]

Exercise and Advice Booklet

Isotonic Exercise Programme

Clifford C, *et al. BMJ Open Sp Ex Med* 2019; 5:e000558. doi: 10.1136/bmjsem-2019-000558

This booklet contains:

- Information about common postures
- The exercises that you should do each day
- An exercise diary

Background Information

The gluteal muscles are a group of muscles located in the region of the buttocks. They attach via a tendon to the bony prominence at the side of the hip. The bony prominence is called the greater trochanter.

The gluteus medius and gluteus minimus tendons are the most commonly injured tendons at the side of the hip. Compression of the gluteal tendons against the greater trochanter can occur during movements of the hip and also during certain postures and positions. This is mainly when the leg crosses the mid-line of the body.

This booklet gives advice and information on how to reduce compression of the gluteal tendons during daily activities and common postures. This is a key component of the exercise programme.

The aims of the exercise programme are to reduce pain and progressively strengthen the gluteal muscles and tendons. Initially you may need to reduce or completely stop some of your normal sports and activities, especially if your symptoms increase significantly afterwards. You can discuss this with a member of the research team.

Clifford C, *et al. BMJ Open Sp Ex Med* 2019; 5:e000558. doi: 10.1136/bmjsem-2019-000558

Common Postures

Lying

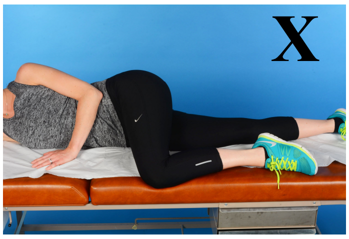

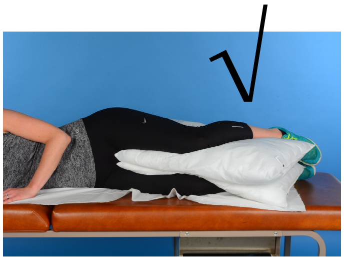

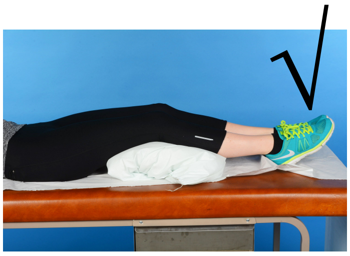

When lying down or sleeping, do not lie on the affected side. If lying on the opposite side, use pillows between your knees to keep leg in parallel position. You can also lie on your back with a pillow under your knees.

Sitting

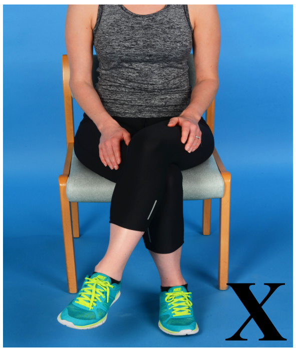

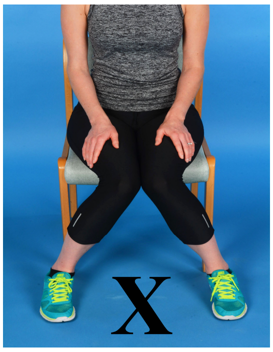

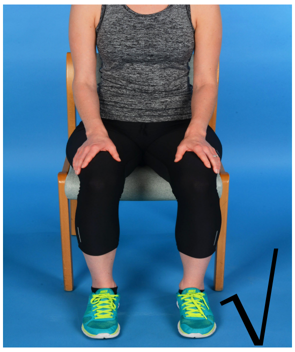

Do not cross your legs or sit with your knees together and feet apart. It may help to sit with your hips higher than your knees, you may need to use a pillow or cushion to do this or if you can, raise your chair.

Standing

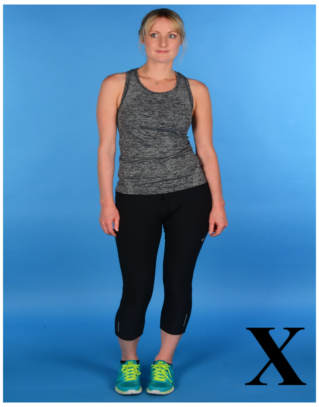

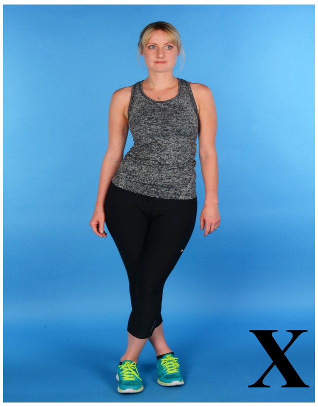

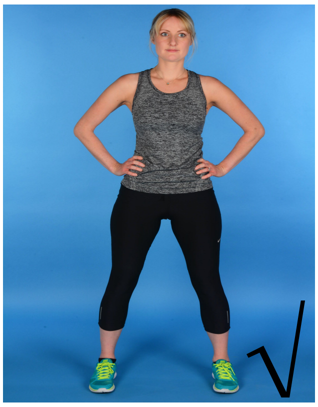

Do not stand ‘hanging on hip’ with all your weight on one leg or stand with your legs crossed. Stand upright with weight evenly shared between both legs and feet shoulder width apart.

Clifford C, *et al. BMJ Open Sp Ex Med* 2019; 5:e000558. doi: 10.1136/bmjsem-2019-000558

Rising from a chair

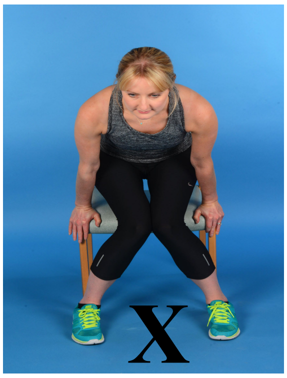

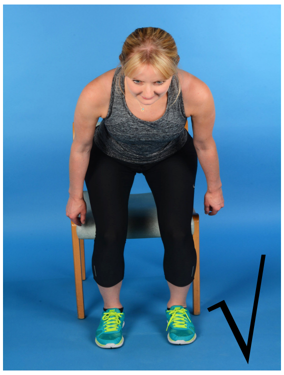

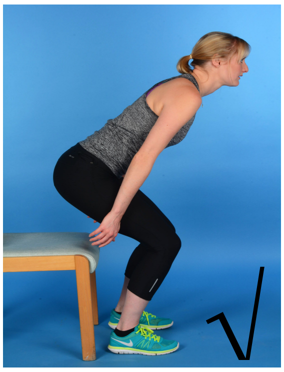

Keep knees apart. Do not allow knees to roll in across body, squeeze buttocks as you stand to help prevent this. Lean forward, bending at your hips and knees while keeping back straight.

Going up stairs

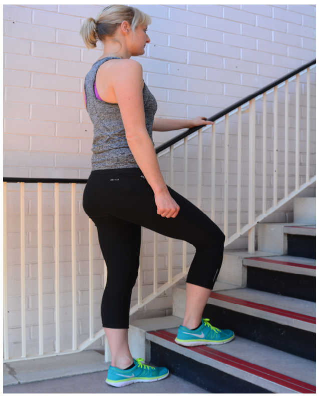
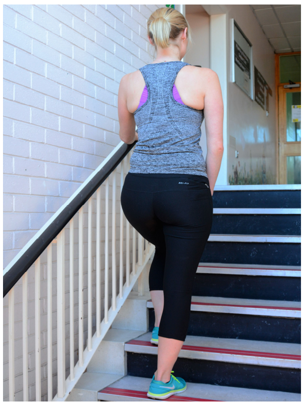

If going up stairs is painful, use a hand rail on the opposite side from your affected leg. Keep feet a little wider.

Stretching

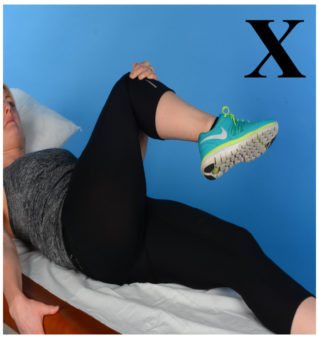

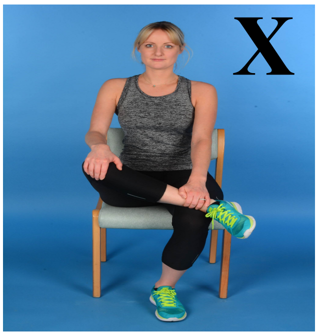

Avoid stretching your leg across your body or stretching your leg by pushing your knee down while the foot is placed on the opposite knee.

Clifford C, *et al. BMJ Open Sp Ex Med* 2019; 5:e000558. doi: 10.1136/bmjsem-2019-000558

Exercises

Page 5 of 21

1. Isotonic Side-Lying Hip Abduction

During isotonic exercise the muscle length and position of the leg change. The muscle will both shorten and lengthen during movement.

Starting position

Lie on your non-affected side. Bend the lower knee. Put 1 or 2 pillows between your knees so that your affected leg doesn't cross the mid-line of your body (Picture 1).

Exercise

Lift the affected leg up slowly to the count of 3 seconds and then slowly lower to the count of 3 seconds (Picture 2). This movement is called hip abduction. Do not allow this knee to bend. Do not allow your pelvis to move backwards.

How often?

Try to complete 3 sets of 10 repetitions. Rest for 60 seconds between each set.

Complete once a day

Progress to using a resistance band around both ankles when advised by your physiotherapist.

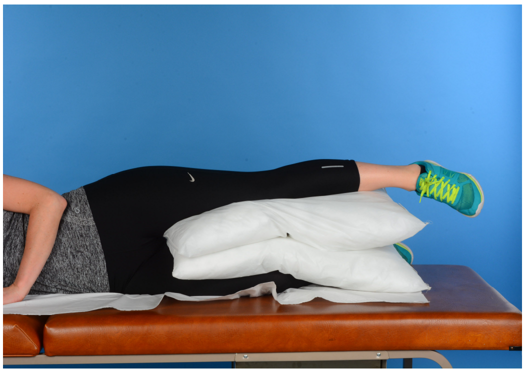

Picture 1

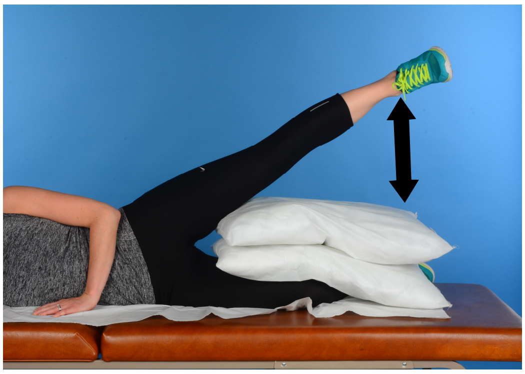

Picture 2

2. Isotonic Standing Hip Abduction Slides

Starting position

Stand with feet slightly wider than shoulder width apart with hands supported on chair or table (Picture 1).

Exercise

Keep your foot in contact with the floor and slide affected leg out to the side to the count of 3 seconds. You can allow the opposite knee to bend but keep your back straight (Picture 2). Slide leg back to starting position to the count of 3 seconds.

How often?

Try to complete 3 sets of 10 repetitions. Rest for 60 seconds between sets.

Complete once a day

Progress to using a resistance band around both ankles when advised by your physiotherapist.

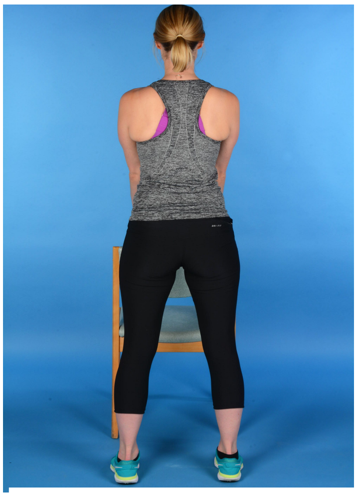

Picture 1

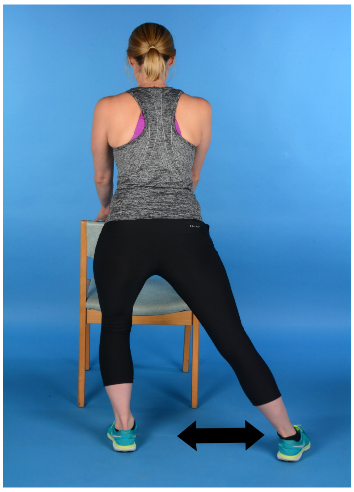

Picture 2

Clifford C, *et al. BMJ Open Sp Ex Med* 2019; 5:e000558. doi: 10.1136/bmjsem-2019-000558

University of Glasgow

NHS Greater Glasgow and Clyde

Pain Monitoring Model

Numerical Pain Rating Scale (NPRS)

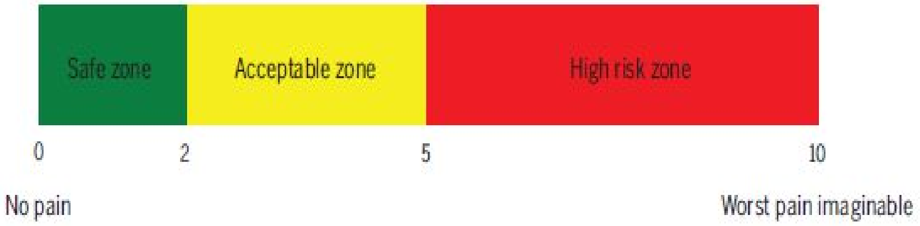

1. The pain is allowed to reach 5 on the NPRS during the activity.
2. The pain after completion of the activity is allowed to reach 5 on the NPRS.
3. The pain the morning after the activity should not exceed a 5 on the NPRS.
4. Pain and stiffness is not allowed to increase from week to week.

Adapted from Silbernagel 2007

You can use this pain scale can be used to monitor your pain during your exercise programme. It ranges from 0 (no pain) to 10 (worse pain imaginable). The pain is allowed to reach 5 during your exercise programme. If the pain you experience during your exercise programme is more than 5, you should reduce the number of repetitions.

Clifford C, *et al. BMJ Open Sp Ex Med* 2019; 5:e000558. doi: 10.1136/bmjsem-2019-000558

Exercise Diary

This should be completed every day for 12 weeks.

Clifford C, *et al. BMJ Open Sp Ex Med* 2019; 5:e000558. doi: 10.1136/bmjsem-2019-000558

**Week 1**	Exercise 1<br>Number of repetitions completed<br>(1-6)	Exercise 2<br>Number of repetitions completed<br>(1-6)	Maximum pain score during exercise programme<br>(1-10)
Day 1			
Day 2			
Day 3			
Day 4			
Day 5			
Day 6			
Day 7			

Clifford C, *et al. BMJ Open Sp Ex Med* 2019; 5:e000558. doi: 10.1136/bmjsem-2019-000558

**Week 2**	Exercise 1<br>Number of repetitions completed<br>(1-6)	Exercise 2<br>Number of repetitions completed<br>(1-6)	Maximum pain score during exercise programme<br>(1-10)
Day 1			
Day 2			
Day 3			
Day 4			
Day 5			
Day 6			
Day 7			

Clifford C, *et al. BMJ Open Sp Ex Med* 2019; 5:e000558. doi: 10.1136/bmjsem-2019-000558

**Week 3**	Exercise 1<br>Number of repetitions completed<br>(1-6)	Exercise 2<br>Number of repetitions completed<br>(1-6)	Maximum pain score during exercise programme<br>(1-10)
Day 1			
Day 2			
Day 3			
Day 4			
Day 5			
Day 6			
Day 7			

Clifford C, *et al. BMJ Open Sp Ex Med* 2019; 5:e000558. doi: 10.1136/bmjsem-2019-000558

**Week 4**	Exercise 1<br>Number of repetitions completed<br>(1-6)	Exercise 2<br>Number of repetitions completed<br>(1-6)	Maximum pain score during exercise programme<br>(1-10)
Day 1			
Day 2			
Day 3			
Day 4			
Day 5			
Day 6			
Day 7			

Clifford C, *et al. BMJ Open Sp Ex Med* 2019; 5:e000558. doi: 10.1136/bmjsem-2019-000558

**Week 5**	Exercise 1<br>Number of repetitions completed<br>(1-6)	Exercise 2<br>Number of repetitions completed<br>(1-6)	Maximum pain score during exercise programme<br>(1-10)
Day 1			
Day 2			
Day 3			
Day 4			
Day 5			
Day 6			
Day 7			

Clifford C, *et al. BMJ Open Sp Ex Med* 2019; 5:e000558. doi: 10.1136/bmjsem-2019-000558

**Week 6**	Exercise 1<br>Number of repetitions completed<br>(1-6)	Exercise 2<br>Number of repetitions completed<br>(1-6)	Maximum pain score during exercise programme<br>(1-10)
Day 1			
Day 2			
Day 3			
Day 4			
Day 5			
Day 6			
Day 7			

Clifford C, *et al. BMJ Open Sp Ex Med* 2019; 5:e000558. doi: 10.1136/bmjsem-2019-000558

**Week 7**	Exercise 1<br>Number of repetitions completed<br>(1-6)	Exercise 2<br>Number of repetitions completed<br>(1-6)	Maximum pain score during exercise programme<br>(1-10)
Day 1			
Day 2			
Day 3			
Day 4			
Day 5			
Day 6			
Day 7			

Clifford C, *et al. BMJ Open Sp Ex Med* 2019; 5:e000558. doi: 10.1136/bmjsem-2019-000558

**Week 8**	Exercise 1<br>Number of repetitions completed<br>(1-6)	Exercise 2<br>Number of repetitions completed<br>(1-6)	Maximum pain score during exercise programme<br>(1-10)
Day 1			
Day 2			
Day 3			
Day 4			
Day 5			
Day 6			
Day 7			

Clifford C, *et al. BMJ Open Sp Ex Med* 2019; 5:e000558. doi: 10.1136/bmjsem-2019-000558

**Week 9**	Exercise 1<br>Number of repetitions completed<br>(1-6)	Exercise 2<br>Number of repetitions completed<br>(1-6)	Maximum pain score during exercise programme<br>(1-10)
Day 1			
Day 2			
Day 3			
Day 4			
Day 5			
Day 6			
Day 7			

Clifford C, *et al. BMJ Open Sp Ex Med* 2019; 5:e000558. doi: 10.1136/bmjsem-2019-000558

**Week 10**	Exercise 1<br>Number of repetitions completed<br>(1-6)	Exercise 2<br>Number of repetitions completed<br>(1-6)	Maximum pain score during exercise programme<br>(1-10)
Day 1			
Day 2			
Day 3			
Day 4			
Day 5			
Day 6			
Day 7			

Clifford C, *et al. BMJ Open Sp Ex Med* 2019; 5:e000558. doi: 10.1136/bmjsem-2019-000558

**Week 11**	Exercise 1<br>Number of repetitions completed<br>(1-6)	Exercise 2<br>Number of repetitions completed<br>(1-6)	Maximum pain score during exercise programme<br>(1-10)
Day 1			
Day 2			
Day 3			
Day 4			
Day 5			
Day 6			
Day 7			

Clifford C, *et al. BMJ Open Sp Ex Med* 2019; 5:e000558. doi: 10.1136/bmjsem-2019-000558

**Week 12**	Exercise 1<br>Number of repetitions completed<br>(1-6)	Exercise 2<br>Number of repetitions completed<br>(1-6)	Maximum pain score during exercise programme<br>(1-10)
Day 1			
Day 2			
Day 3			
Day 4			
Day 5			
Day 6			
Day 7			

Clifford C, *et al. BMJ Open Sp Ex Med* 2019; 5:e000558. doi: 10.1136/bmjsem-2019-000558
